# The Case of Mrs. Crook

**Published:** 1900-03-17

**Authors:** 


// The Hospital

A Journal of

The Medical Sciences and Hospital Administration.

Vol. XXVII.—No. 703.] EDITED BY SIR HENRY BURDETT, K.C.B., AND SOLOMON C. SMITH, M.D., M.R.C.P. [March 17, 1900.

### The Case of Mrs. Crook.

Notwithstanding the admitted excellence of the English system and methods of jurisprudence when regarded as a whole, notwithstanding the uprightness and impartiality of our judges, the skill and fairness of the Bar, and the unimpeachable rectitude of the average jury, there arise, every now and then, matters of contention with which the ordinary tribunals are manifestly ill-adapted to deal, and in which miscarriages of justice, even if they do not actually occur, can only be said to have been avoided by accident. A striking example of this kind has lately occupied the Divorce Court; and, by reason of the circumstance that both the petitioning husband and the co-respondent were members of the medical profession, while the latter occupies a distinguished position as a hospital physician and teacher, has attracted no small amount of attention, alike to the facts which were proved in evidence and to the inferences which might legitimately be drawn from them. What is indisputable is that Mrs. Crook, the young wife of a young medical man, who had been a student at Guy's Hospital, gave birth to her first child in May, 1898, and subsequently suffered from some ailment the nature of which was not disclosed in the report, but for which, at her husband's desire, she went to consult his former teacher, Dr. Horrocks, in the following November. Dr. Horrocks had heard of her from her husband, but he had never seen her before her professional visit to him. She went to his consulting-room in the ordinary way, and went, it was said, "four or five times." It seems probable that Dr. Horrocks, knowing her to be the wife of an old pupil, treated her with somewhat more friendliness than he might have displayed to a stranger, for he showed her some part of his house, and on one occasion helped her on her road home by driving her to Victoria Station in his carriage, and by giving her a book to read on her journey. After the four or five visits the attendance ceased, and Dr. Horrocks did not see the lady again. In the summer of 1899 the petitioner noticed that his wife was "absent-minded," and he himself went in July to consult a "palmist," whose statements made such an impression upon him that he urged his wife to consult her also. Mrs. Crook went to the "palmist" on August 3rd, and seems to have heard from her some abominable talk about being rendered a widow by divorce, and about having a child which would not be her husband's. This preyed upon her mind, and in the following November she made what was described as a "confession," to the effect that she had committed adultery with Dr. Horrocks every time she had visited him, even including the first. Dr. Savage, who was consulted about the case, made the suggestion, at once scientific and sensible, that the lady was the subject of a delusion; but neither science nor common sense could be expected to exert much influence over a medical man who was not ashamed to confess that he had supported so vulgar and so trumpery an imposture as "palmistry," and Dr. Horrocks, who had presumably forgotten all about the case, was unpleasantly reminded of it by being placed in the position of a co-respondent. Beyond the "confession," there was not a scrap of evidence of any kind, and he need only have asked that the petition as against him should be dismissed; but wisely and properly took the bolder course of going into the witness-box and of absolutely denying the truth of the lady's statement. The jury found unanimously, of course, that he had not committed the adultery with which he was charged; but they were unable to arrive at similar unanimity with regard to the lady herself, and were said to be almost equally divided. Some of them thought, it may be supposed, that a husband ought to be released from a wife who, if not actually an adulteress, was yet the subject of adulterous delusions, and that the mental offence was as criminal as the physical act. There can be little doubt that, if the case had been submitted to a jury of experts, of medical men conversant with disordered conditions of the nervous system, and capable of bringing out by cross-examination any relevant matter which might have been furnished either by the domestic life of the petitioner and the respondent, or by the character of the ailment from which the latter had been suffering, very little difficulty would have been experienced by them in arriving at a positive conclusion. It would not have been impossible to organise such a tribunal under the form of an arbitration, with the probable result of shielding the respondent from the sad consequences which can hardly fail to attend upon the publicity given to her so-called confession. It seems surprising that neither Dr. Rowe, who attended the lady in her confinement, nor the solicitors employed on either side, appear to have suggested this simple method of arriving at a solution of the problem, or to have seen that the case was essentially medical in all its aspects. We congratulate Dr. Horrocks on the manner in which he met the charge, and upon the way in which it was dealt with both by judge and jury, but at the same time we must express our doubts about the propriety of medical men seeing lady patients alone if they so desire. It did not appear from the reports whether the interviews with Mrs. Crook were merely conversational or whether some form of physical examination was required, but, in the latter case especially, we feel that the wise rule is for the practitioner to insist upon the presence either of a female friend of the patient's or of a nurse or other suitable attendant employed by himself.

